# M. Jobert’s Method of Treating Fractures

**Published:** 1848-03

**Authors:** 


*M. Jobert's Method of Treating Fractures.*—For the last fourteen years, M. Jobert has treated fractures of the leg, I may say, without the bandage. It would be strange, indeed, if a man of his intelligence, who in more than one respect is in advance of his countrymen, should persist in any plan of treatment, for so many years, if it were not at least equal, and on some accounts superior, to the more common, and, as I might say, more orthodox systems. Placing the fractured member upon a cushion, he embraces the foot with a simple bandage, in the form of a stirrup, the ends of which serve to effect extension, which is afterwards rendered permanent by their being firmly attached to the foot of the bed. Counter-extension is practiced by means of a towel, the centre of which is placed upon the pelvis, and the two extremities fastened to the head of the bed. In this way the limb is ready for the application of topical remedies if rendered necessary, either by a wound of the integuments, a phlegmon, or any other affection. This, however, according to Jobert, is not the only advantage of this method of treatment. The fractured limb, not being submitted to any compression, its circulation is performed as in the normal state ; and certainly it cannot be denied that this is a condition in every way favorable to the prompt formation of the callus. Moreover, in the leg, the muscular powers act but little, and a feeble force is sufficient to maintain the osseous fragments in a position suitable for obtaining a consolidation without shortening.

I do not propose to compare this mode of treatment of fractures of the leg with those employed by other surgeons. It is at any rate simple, and any who will follow M. Jobert in his visits will see, among probably the largest number of fractures that are to be found in any service in Paris, the most perfect and beautiful cures of this class of accidents effected by a strip of bandage and a towel. —*Foreign Correspondence of Western Jour. of Med. and Sur.*

